# The Instigation of the Associations Between Melatonin, Circadian Genes, and Epileptic Spasms in Infant Rats

**DOI:** 10.3389/fneur.2020.497225

**Published:** 2020-10-26

**Authors:** Lin Wan, Xiu-Yu Shi, Wen-Rong Ge, Yu-Lin Sun, Shan Zhang, Jing Wang, Lin-Yan Hu, Li-Ping Zou, Guang Yang

**Affiliations:** ^1^The First Medical Center of the Chinese People's Liberation Army General Hospital, Beijing, China; ^2^Beijing Friendship Hospital, Capital Medical University, Beijing, China

**Keywords:** infantile spasm, circadian rhythm, circadian gene, adrenocorticotropic hormone, melatonin

## Abstract

**Background:** Infantile spasm (IS) is one of the most common catastrophic epilepsy syndromes in infancy characterized by epileptic spasm. While adrenocorticotropic hormone (ACTH) is the first-line treatment for IS, it is evident that the seizures associated with IS exhibit a clear circadian rhythm; however, the precise mechanisms underlying such seizures remain unclear. Melatonin is an important amine hormone and is regulated by circadian rhythm. Circadian proteins, especially Aryl Hydrocarbon Receptor Nuclear Trasnslocator-like Protein (ARNTL or BMAL1) and Circadian Locomotor Output Cycles Kaput (CLOCK), and their target proteins Period Circadian Regulator 1 (PER1), Period Circadian Regulator 2 (PER2), Cryptochrome 1 (CRY1), and Cryptochrome 2 (CRY2), play key roles in circadian rhythm. This study explored the relationships between melatonin, genes associated with circadian rhythm, and epileptic spasm.

**Materials and Methods:** Eighteen female rats were mated with nine male rats and 16 became pregnant. Twelve pregnant rats were subjected to prenatal stress by forced swimming in cold water from the day of conception. Rat pups produced by stressed mothers received an intraperitoneal injection of N-methyl-D-aspartate (NMDA) on the 13th day after birth and were divided into four groups: NMDA (15 mg/kg), NMDA+ACTH (20 IU/kg), NMDA+melatonin (55 mg/kg), and NMDA+ACTH+melatonin (*n* = 36/group). Offspring from four dams that were not subjected to prenatal stress were used as controls. We then recorded latency and the frequency of flexion seizures. All offspring were sacrificed on the 14th day after birth and CLOCK, BMAL1, PER1, PER2, CRY1, and CRY2 expression was analyzed by western blotting, immunohistochemistry, and immunofluorescence.

**Results:** NMDA induced spasm-like symptoms in rats. ACTH and melatonin significantly increased seizure latency and significantly reduced the frequency of seizures (*P* < 0.05). CLOCK, BMAL1, PER1, PER2, CRY1, and CRY2 expression was significantly lower in the NMDA group than the controls (*P* < 0.05). ACTH significantly increased the expression of CLOCK, BAML1, PER1, and CRY1 (*P* < 0.05) and melatonin significantly increased the expression of CLOCK, BMAL1, PER1, PER2, CRY1, and CRY2 (*P* < 0.05) compared with those of the NMDA group. There were no significant differences in the expression of BMAL1, CRY2, PER1, and PER2 when compared between the NMDA+ACTH+melatonin and control groups (*P* > 0.05).

**Conclusion:** ACTH and melatonin significantly increased the expression of circadian genes and improved NMDA-induced seizures. The anticonvulsant effects of ACTH and melatonin are likely to involve regulation of the expression of these genes.

## Introduction

Infantile spasm (IS) is a catastrophic and age-specific epilepsy syndrome characterized by epileptic spasm that occurs in infancy and can exert severe effects on growth and development. IS seizures are associated with circadian rhythm and mostly occur when infants experience arousal conditions and during the daytime, particularly during early drowsiness or after awakening; seizures are very rare during sleep (1–4). The specific pathogenesis of IS has yet to be fully elucidated although previous studies have suggested that it may be associated with dysfunction of the hypothalamic-pituitary-adrenal (HPA) axis (5). Adrenocorticotropic hormone (ACTH), as an intermediate product of the HPA axis, and because of its ability to reduce levels of corticotropin-releasing hormone (CRH) via negative feedback, is commonly used as a first-line treatment for IS; however, the precise mechanism involved remains unclear. The HPA axis is known to be closely related to the circadian rhythm. For example, a number of studies have demonstrated a circadian rhythm in the neuronal regulation of the suprachiasmatic nucleus in the hypothalamus (6–8). Based on such findings, it is possible that the pathogenesis of IS may be related to the excessive release of CRH in the hypothalamus. Previous research has demonstrated a close relationship between ACTH and circadian rhythm (9–11). However, whether ACTH can alter the expression levels of genes responsible for the circadian rhythm in IS, and whether its anticonvulsant effects are related to the regulation of circadian rhythm genes, remains unclear.

Circadian Locomotor Output Cycles Kaput (CLOCK), Aryl Hydrocarbon Receptor Nuclear Trasnslocator-like Protein (ARNTL or BMAL1), Period Circadian Regulator 1 (PER1), Period Circadian Regulator 1 (PER2), Cryptochrome 1(CRY1), and Cryptochrome (CRY2) are considered to be the core proteins responsible for the circadian rhythm. These proteins interact with each other to influence the transcription of other circadian rhythm genes which mediate various physiological and pathological processes in the nervous system (12, 13).

Several studies have shown that epilepsy is closely related to the circadian rhythm. For example, Quigg et al. (14) reported obvious circadian rhythms in some types of epileptic seizures in both humans and experimental mice. In another study, Durazzo et al. (15) retrospectively analyzed the electroencephalogram records of 131 adults with epilepsy and found that most epileptic seizures originating from the occipital and temporal lobes occurred in the afternoon, while seizures originating from the frontal and parietal lobes usually occurred at night. These findings indicated that the endogenous circadian rhythm differentially influences the manifestations of epilepsy in different regions of the brain (15). However, whether the pathogenesis of IS is related to abnormalities in factors associated with the circadian rhythm has yet to be fully elucidated.

Melatonin is an amine hormone synthesized and secreted by the pineal gland, which is regulated by the circadian rhythm and can also exert effects upon the circadian rhythm. The secreted levels of melatonin during the night can be 5–10 times higher than that during the daytime; consequently, melatonin can be used as a drug for sleep disorders to regulate circadian rhythms in patients with insomnia (16–18). Previous studies have shown that epilepsy is associated with melatonin levels. For example, the administration of exogenous melatonin can reduce the number of seizures, prolong the latency to seizure and reduce the severity of epilepsy (19, 20). In a previous study, Mosińska et al. (21) suggested that melatonin may control the frequency and threshold of epileptic seizures by regulating the expression of circadian rhythm genes. However, the precise mechanism underlying this protective effect, and whether melatonin can restore the expression of circadian rhythm genes in IS, has yet to be elucidated.

There are mainly three ways to induce the acute animal model of infantile spasm, such as NMDA rat model, prenatal betamethasone/stress+post-natal NMDA rat model and GBL/Down syndrome mouse model. We choose the prenatal stress+post-natal NMDA rat model for its characteristics with age dependency, spasm-like seizures, cognitive impairment and response to ACTH, which fulfills the criteria of an infantile spasm model (22).

The aim of this study was to observe the role of the circadian rhythm in the pathogenesis of epileptic spasm and to investigate whether the anticonvulsant effects of ACTH and melatonin are related to the regulation of circadian rhythm factors in an N-methyl-D-aspartate (NMDA)-induced infant rat model of acute spasm.

## Materials and Methods

### Animals and Prenatal Treatment

Nine 3-month-old male Sprague–Dawley (SD) rats, and 18 female SD rats, weighing 270–350 g, were selected from the Laboratory Animal Center of Chinese PLA General Hospital and placed in a standard polycarbonate cage (35 × 30 × 17 cm) at a temperature of 20°C, a relative humidity of 50–60% and with a controlled photoperiod (12 h light:12 h dark; lights on and off at 8 am and 8 pm, respectively). Food and drinking water were provided *ad libitum* and the rats were allowed to acclimatize for 1 week prior to experimentation.

Female rats were then mated with male rats. The presence of a vaginal plug was considered as gestational day 1; in total, 16 female rats became pregnant. From gestational day 1, 12 pregnant dams were subjected to stress. To induce stress, the pregnant rats were placed into a transparent plexiglass bucket (50 cm in height × 20 cm in diameter) filled with cold water (4°C) at 5 pm each day and forced to swim for 5 min. Thereafter, the rats were removed from the bucket and dried in a heated container for 10 min before being returned to the cage. Stress was administered daily until parturition. The other four dams were not exposed to stress. We ensured that we used the minimum number of animals necessary and took great care to reduce any unnecessary discomfort. This study was approved by the Ethics Committee of the First Medical Center of the PLA General Hospital, China (Reference number: 20160134).

### Experimental Groups

Offspring without prenatal stress were used as a control group (*n* = 36). The offspring exposed to prenatal stress (*n* = 144) were divided into four groups (*n* = 36 per group): (1) NMDA group, in which rats were injected intraperitoneally (i.p.) with NMDA (15 mg/kg); (2) NMDA+ACTH group, in which rats were injected (i.p.) with ACTH (20 IU/kg) 30 min before NMDA administration; (3) NMDA + melatonin (MLT) group, in which rats were given melatonin (55 mg/kg) 30 min before NMDA administration; and (4) NMDA+MLT+ACTH group, in which rats were injected (i.p.) with ACTH (20 IU/kg) and melatonin (55 mg/kg) 30 min before NMDA administration.

### NMDA Rat Model

The day of birth was considered as post-natal day 1 (P1). On P13, all offspring exposed to prenatal stress (*n* = 144) were injected (i.p.) with NMDA (15 mg/kg) to induce epileptic seizures. We then recorded the latency from injection to flexion seizure and the number of flexion seizures. The severity of seizures was evaluated according to an established system: grade 0 (no seizures, normally active); grade 1 (stable and puffing); grade 2 (hyperactive, irritated, twisting tail continuously); grade 3 (bothering itself and others); grade 4 (repeated flexion, *n* ≤ 4); grade 5 (repeated flexion, 5 ≤ *n* ≤ 14); grade 6 (repeated flexion, 15 ≤ *n* ≤ 29); grade 7 (repeated flexion, *n* ≥ 30); grade 8 (extremities showing thrashing-like movements after tonic–clonic seizures); and grade 9 (death). All rats were continuously monitored for 3 h following the injection of NMDA.

### Detection of Factors Associated With Circadian Rhythm

All experimental and control rats were sacrificed with a ketamine/cilazine cocktail 24 h after the last injection of drugs, such as NMDA, ACTH, and/or melatonin. We then removed the hypothalamus from each rat.

#### Western Blot Analyses

Western blot analyses were used to identify hypothalamic tissue (*n* = 9/group). Tissue extracts were washed three times in phosphate-buffered saline (PBS, pH 7.0) prior to the addition of 1 mL radioimmunoprecipitation assay (RIPA) buffer (Beyotime, Shanghai, China) containing a protease inhibitor cocktail (Beyotime, Shanghai, China). This was then incubated for 30 min and then centrifuged at 12,000 *g* for 10 min at 4°C. The supernatant was then removed and stored in aliquots at −80°C to await further analysis. Protein concentration was measured using the Bradford assay (23). For each rat, an equal amount of protein extract (20–50 μg) was then separated by 7–12% sodium dodecyl sulfate (SDS) polyacrylamide gel electrophoresis and transferred electrophoretically to polyvinylidene difluoride membranes (Millipore, Billerica, MA, USA). The membranes were then blocked with 5% skimmed milk containing 0.05% Tween 20 with Tris-buffered saline (TBST) for 2 h at 37°C and then incubated overnight at 4°C with a range of antibodies against BMAL1, CLOCK, PER1 (1:300; Abcam, Cambridge, MA, USA), PER2, CRY2 (both rabbit antibodies; 1:1,000; Thermo Fisher Scientific, Waltham, MA, USA) and CRY1 (mouse antibody; 1:500; Santa Cruz Biotechnology, CA, USA). Antibodies were diluted in TBST containing 2.5% skimmed milk. A rabbit anti-glyceraldehyde 3-phosphate dehydrogenase (GAPDH) polyclonal antibody (1:1,000; Xianzhi Biotechnology, Hangzhou, China) was used to demonstrate equal protein loading. The next day, the membranes were incubated with a horseradish peroxidase (HRP)-conjugated anti-mouse or anti-rabbit secondary antibody (1:5,000; Boster Biological Technology, Wuhan, China) for 2 h at 37°C and visualized using an enhanced chemiluminescence detection kit (Thermo Fisher Scientific, Waltham, MA, USA). Band intensities were quantified using ImageJ software v3.91 (NIH, Bethesda, MD, USA) and each sample was normalized according to GAPDH.

#### Immunohistochemical Staining

Hypothalamic tissue samples (*n* = 3/group) were fixed in 10% buffered formalin and embedded in paraffin wax. Sections (4 μm) were then cut and then dewaxed and dehydrated using a graded series of ethanol concentrations. Sections were then covered in 25 mM citrate buffer (pH 6.0), boiled and then allowed to cool for 40 min. Hydrogen peroxide (3%) was used to inactivate endogenous peroxidase activity. Sections were then blocked and permeabilized with normal goat serum (Abcam, Cambridge, MA, USA) for 30 min at room temperature. Next, sections were incubated overnight at 4°C with rabbit antibodies against BMAL1 and CLOCK (1:100; Abcam, Cambridge, MA, USA) and a mouse antibody against CRY1 (1:100; Santa Cruz Biotechnology, CA, USA). The next day, sections were incubated with an HRP-conjugated anti-mouse or anti-rabbit secondary antibody (Boster Biological Technology, Wuhan, China) for 30 min. Color was developed with a solution of 3–3-diaminobenzidine (Dako Denmark A/S, Glostrup, Denmark), and the results were assessed with a Dako REAL EnVision Detection System. The specificity of antibody binding was evaluated using negative controls (incubation with PBS without antibody). Finally, areas showing positive antibody binding were quantified using ImageJ software v3.91 (NIH, Bethesda, MD, USA).

#### Immunofluorescence Staining

**Figure 1:**
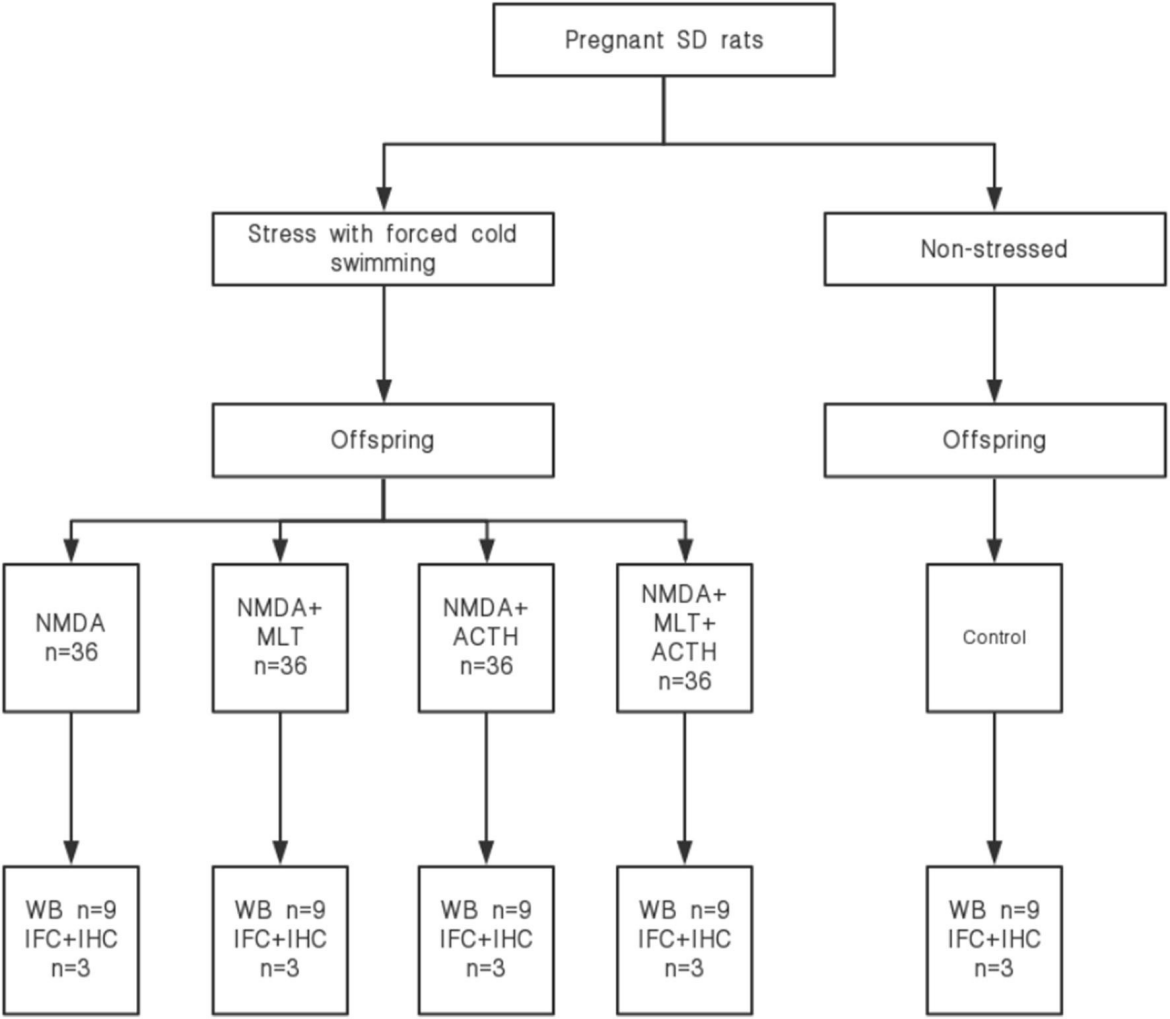
Schematic showing the experimental protocol.

For immunofluorescence staining, sections (4 μm) of hypothalamic tissue (*n* = 3/group) were dewaxed and hydrated using a graded series of ethanol concentrations, boiled in 25 mM citrate buffer (pH 6.0) for 10 min and then cooled with cold deionized water for 1 h. Sections on coverslips were then washed three times in PBS and blocked and permeabilized with normal goat serum (Abcam, Cambridge, MA, USA) for 30 min at room temperature. After drying, sections were incubated overnight at 4°C with rabbit antibodies against BMAL1 and CLOCK (1:100; Abcam, Cambridge, MA, USA) and a mouse antibody against CRY1 (1:100; Santa Cruz Biotechnology, CA, USA). All antibodies were used at a dilution of 1:100. The next day, sections were washed three times in PBS and then incubated with Cy3-conjugated goat anti-rabbit IgG secondary antibody and Cy3-conjugated goat anti-mouse IgG secondary antibody (Boster Biological Technology, Wuhan, China) for 1 h at 25°C. Coverslips were then washed three times with PBS, mounted on glass slides with Fluoromount-G (SouthernBiotech, Birmingham, AL, USA) with 4′,6-diamidino-2-phenylindole (DAPI; Beyotime; Shanghai; China) and visualized using a BX53 microscope (Olympus, Tokyo, Japan). Areas of positive antibody binding were then quantified using ImageJ software v3.91 (NIH, Bethesda, MD, USA). A schematic diagram of the experimental process is shown in Figure 1.

### Data Analysis

Results are expressed as mean ± standard error of the mean (SEM). Data were first evaluated using the Kolmogorov–Smirnov test to determine whether the data were normally distributed. Normally distributed data were then tested for significance using independent-sample *t-*tests. Analysis of variance (ANOVA) was used to test the significance of data that were not normally distributed. The correlations between the expression of factors associated with circadian rhythm and seizure frequency and latency of all the rats with spasms were analyzed using Spearman's correlation coefficient. All statistical analyses were carried out using SPSS 22.0 (IBM Corp., Armonk, NY, USA). *P* < 0.05 was considered statistically significant.

## Results

### NMDA Injection Induced Spasm-Like Seizures in Rats

**Figure 2:**
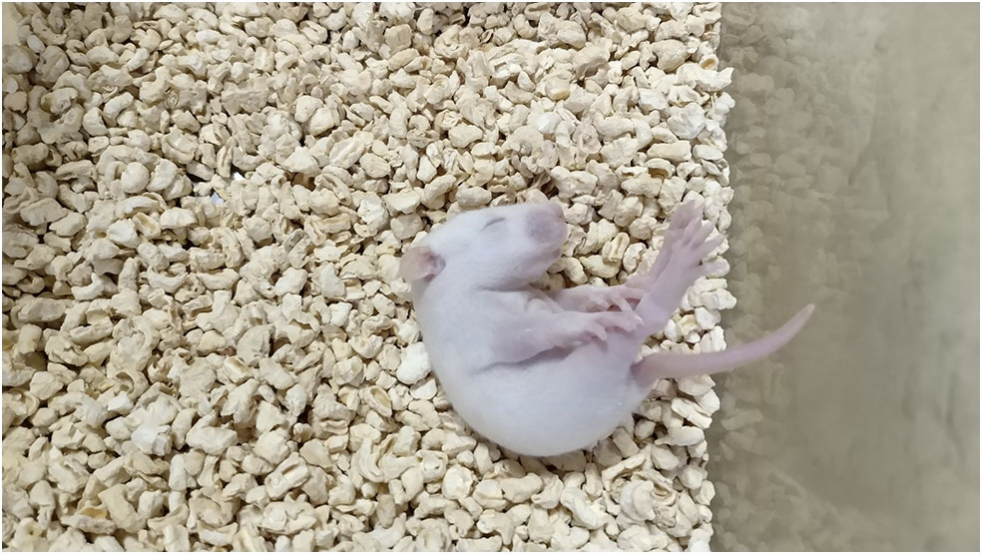
The injection of NMDA induced spasm-like seizures.

**Figure 3:**
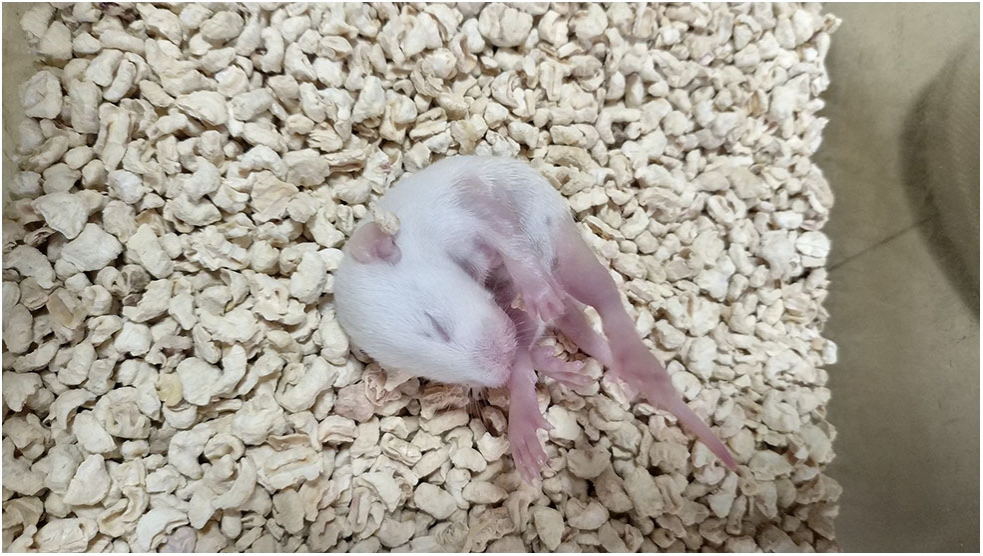
The injection of NMDA induced spasm-like seizures.

The intraperitoneal injection of NMDA into rat pups induced spasm-like symptoms. The specific characteristics of curling spasms, in which rats curled into a ball-like shape, included spine curling, the head meeting the tail, the paws curling against the body or the entire body forming a ball-like shape. Spasms occurred singularly or continuously. We also observed tonic seizures and even death (Figures 2, 3).

### Effects of Melatonin and ACTH on NMDA-Induced Seizures

In the NMDA group, the mean latency to the onset of flexion was 25.91 ± 3.53 min, and the mean number of flexion seizures was 15.26 ± 2.44. In the NMDA+ACTH group, the administration of ACTH significantly increased the latency to flexion seizure (39.47 ± 2.86 min) (*P* < 0.001) and significantly reduced the number of flexion seizures (9.01 ± 1.71) (*P* < 0.001). In the NMDA+MLT group, the administration of melatonin also significantly increased the latency to flexion seizure (37.13 ± 3.92 min) (*P* < 0.001) and significantly reduced the number of flexion seizures (11.29 ± 3.79) (*P* < 0.001). The combined effect of ACTH and melatonin was also significant (*P* < 0.001, Table 1) and was much more obvious.

**Table 1:** Effects of ACTH and melatonin on seizure.

	**Latency (min)**	***P***	**Seizures (number)**	***P***
NMDA group	25.91 ± 3.53	–	15.26 ± 2.44	–
NMDA+ACTH group	39.47 ± 2.86	<0.001	9.09 ± 1.71	<0.001
NMDA+MLT group	37.15 ± 3.92	<0.001	11.29 ± 3.79	<0.001
NMDA+ACTH+MLT group	42.82 ± 2.50	<0.001	6.79 ± 2.16	<0.001

### ACTH and Melatonin Altered the Expression of Circadian Rhythm Factors

**Figure 4:**
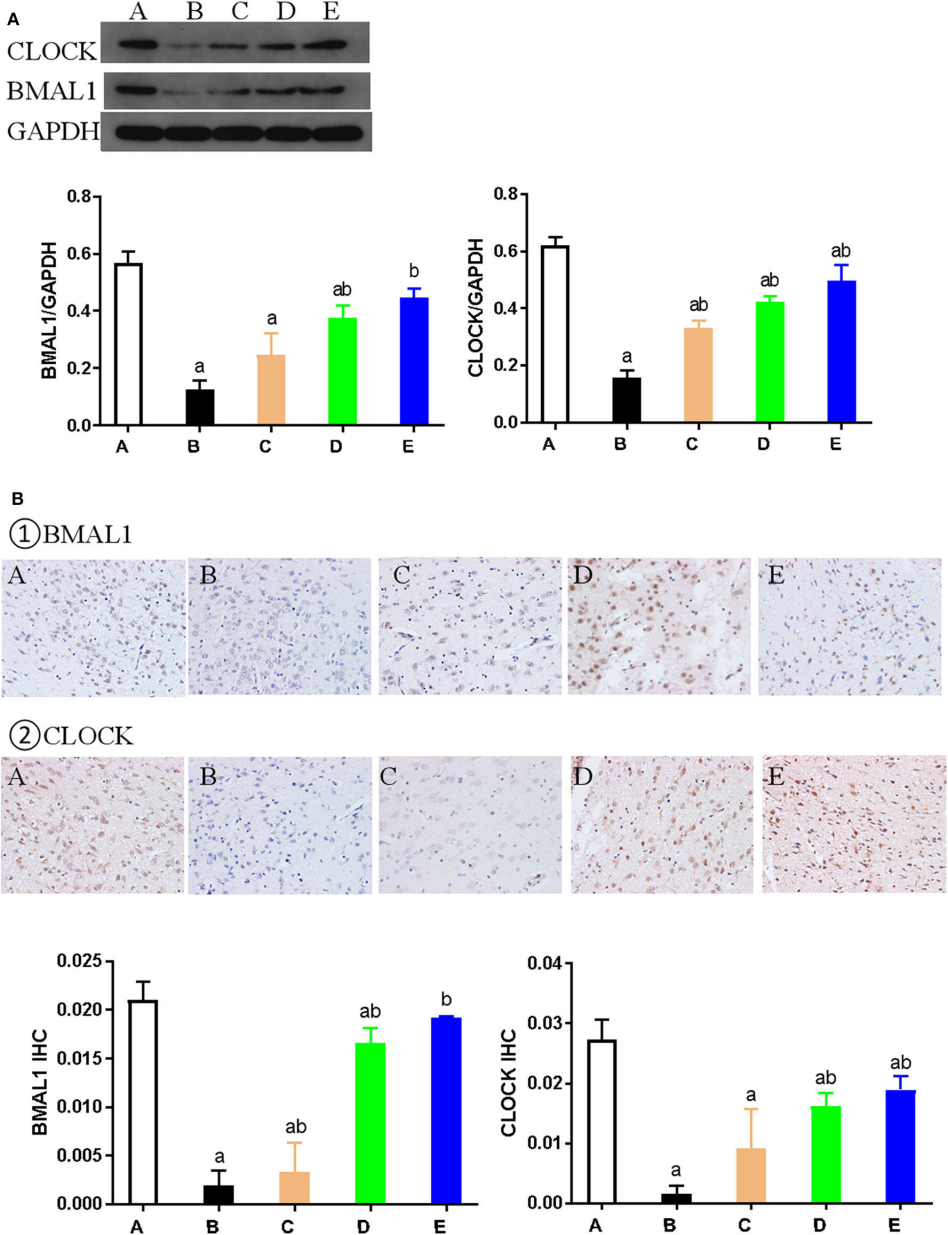
Expression of CLOCK and BMAL1 proteins in each group. **(A)** Representative western blot images and densitometric quantification of CLOCK and BMAL1 proteins from hypothalamus extracts. The equal loading of proteins is illustrated by GAPDH. Values are expressed as mean ± standard error of the mean (SEM; *n* = 9). **(B)** Hypothalamus CLOCK and BMAL1 immunohistochemistry. Paraffin-embedded sections were stained with antibodies against CLOCK and BMAL1. Original magnification: ×400. Values are expressed as mean ± SEM of experiments performed in triplicate. A: control group; B: NMDA group; C: ACTH group; D: MLT group; E: ACTH+MLT group. ^*a*^*P* < 0.05 compared with control group; ^*b*^*P* < 0.05, compared with NMDA group.

Western blotting was used to detect the expression of key proteins associated with the circadian rhythm. The expression of several circadian rhythm factors (CLOCK, BMAL1, PER1, PER2, CRY1, and CRY2) in the NMDA group was significantly lower than in the control group (*P* < 0.05). However, compared with the NMDA group, ACTH treatment led to a significant increase in the expression of CLOCK, BMAL1, PER1, and CRY1 (*P* < 0.05). Additionally, compared with the NMDA group, the expression of CLOCK, BMAL1, PER1, PER2, CRY1, and CRY2 all increased significantly (*P* < 0.05) in the NMDA+ACTH+MLT and NMDA+MLT groups. Furthermore, there were no significant differences in the expression of BMAL1, CRY2, PER1, and PER2 between the NMDA+ACTH+MLT and control groups (*P* > 0.05) (Table 2, Figures 4A, 6A).

**Table 2:** Expression of circadian rhythm proteins.

	**Control group**	**NMDA group**	**NMDA+ACTH group**	**NMDA+MLT group**	**NMDA+ACTH+MLT group**
CRY1/ GAPDH	0.786 ± 0.075	0.141 ± 0.011^a^	0.251 ± 0.031^a^^b^	0.39 ± 0.049^a^^b^	0.578 ± 0.028^a^^b^
CRY2/ GAPDH	0.605 ± 0.047	0.118 ± 0.019^a^	0.237 ± 0.066^b^	0.381 ± 0.076^a^^b^	0.505 ± 0.066^b^
PER1/ GAPDH	0.653 ± 0.081	0.142 ± 0.027^a^	0.276 ± 0.038^a^^b^	0.422 ± 0.046^a^^b^	0.549 ± 0.07^b^
PER2/ GAPDH	0.789 ± 0.128	0.177 ± 0.062^a^	0.332 ± 0.085^b^	0.479 ± 0.063^a^^b^	0.623 ± 0.11^b^
CLOCK/ GAPDH	0.621 ± 0.027	0.16 ± 0.024^a^	0.332 ± 0.025^b^	0.424 ± 0.019^a^^b^	0.50 ± 0.055^b^
BMAL1/ GAPDH	0.569 ± 0.038	0.126 ± 0.031^a^	0.246 ± 0.076^a^^b^	0.376 ± 0.043^a^^b^	0.446 ± 0.033^b^

a *P < 0.05 compared with control group*;

b *P < 0.05 compared with NMDA group*.

**Figure 5:**
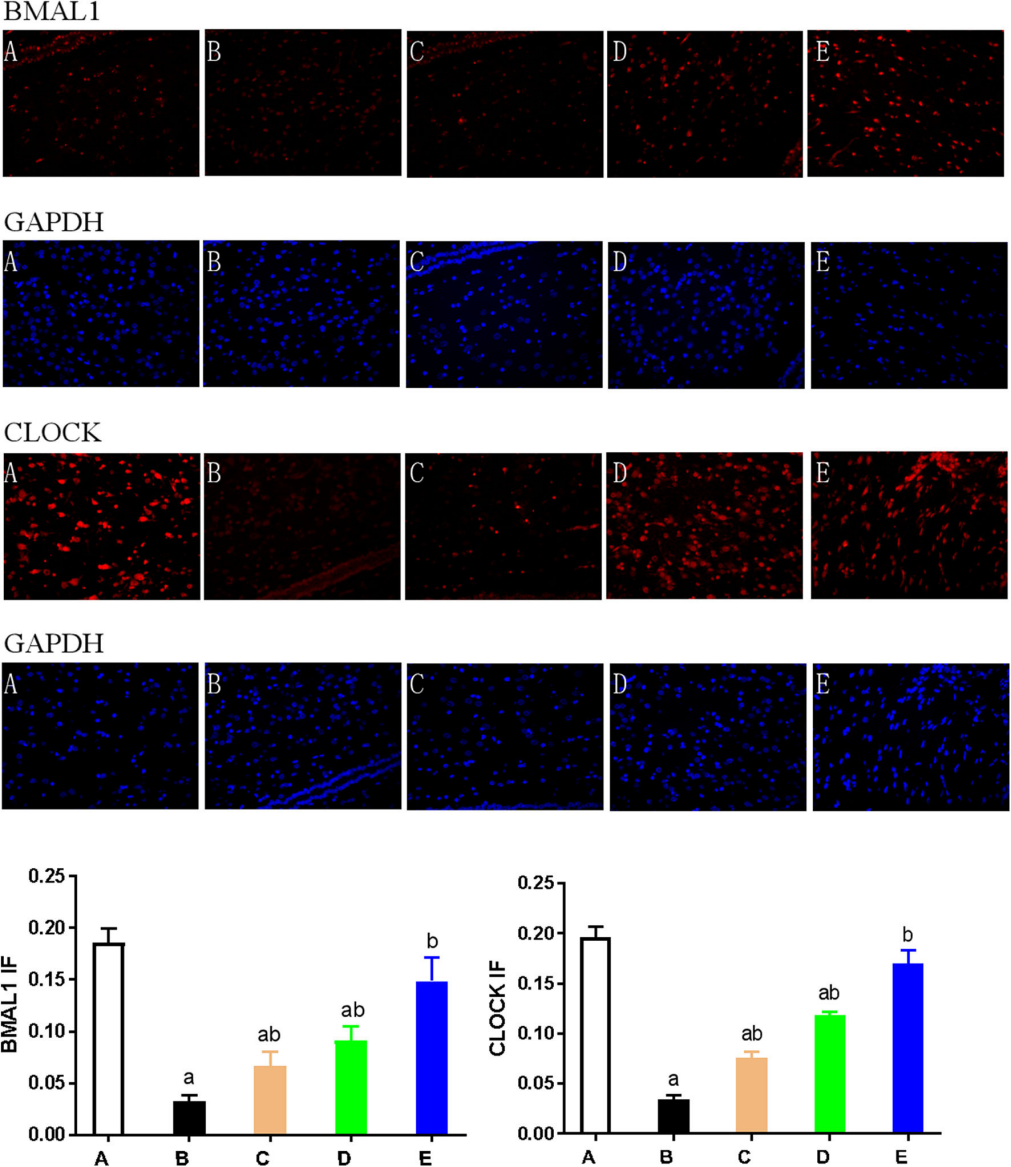
Expression of CLOCK and BMAL1 as detected by immunofluorescence. Hypothalamus tissue was stained with antibodies against BMAL1 and CLOCK (red). DAPI staining (blue) denotes cell nuclei. Values are expressed as mean ± standard error of the mean (SEM) of experiments performed in triplicate. A: control group; B: NMDA group; C: ACTH group; D: MLT group; E: ACTH+MLT group. ^*a*^*P* < 0.05 compared with the control group; ^*b*^*P* < 0.05 compared with the NMDA group.

**Figure 6:**
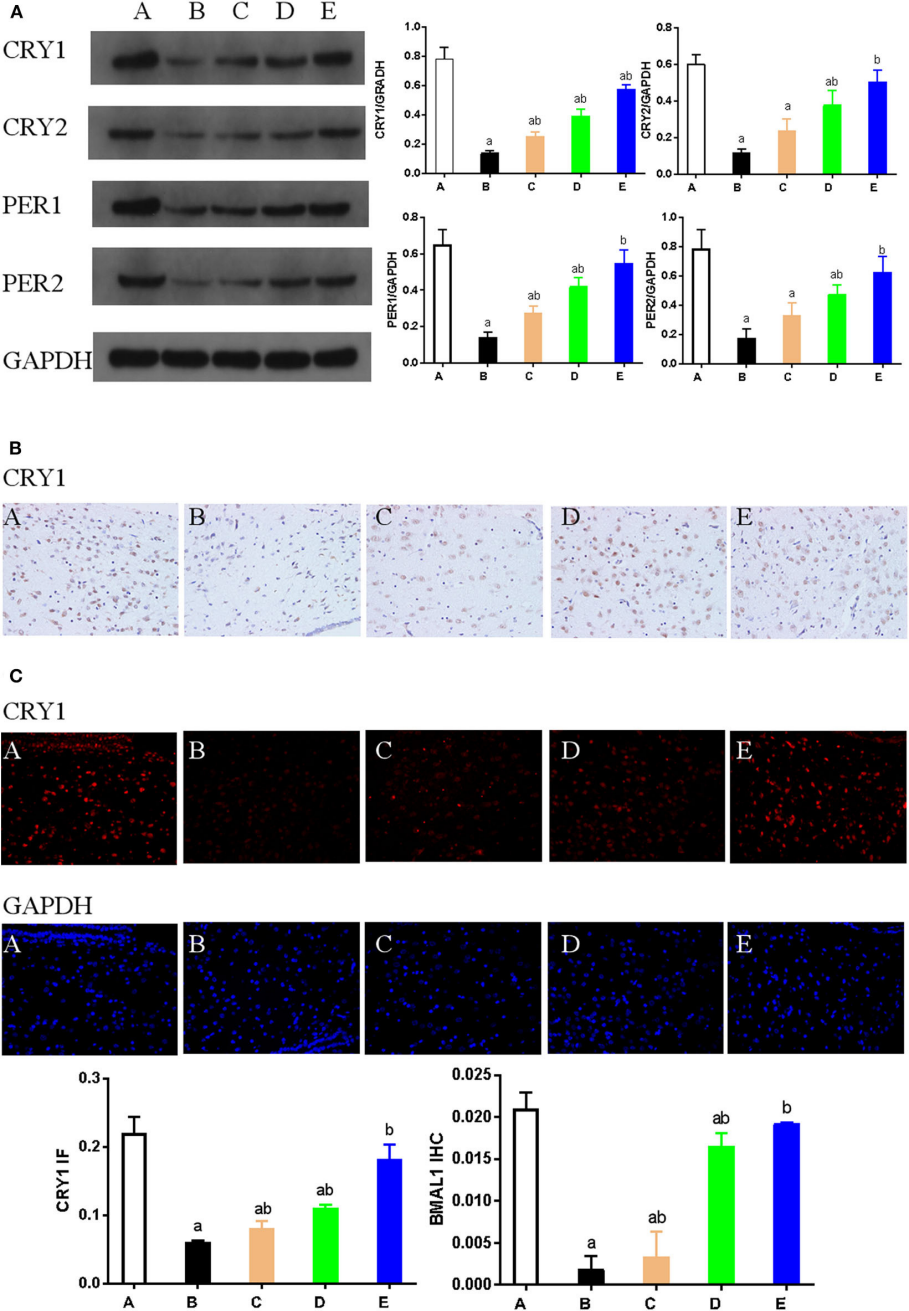
**(A)**. Representative western blot images and densitometric quantification of PER1, PER2, CRY1, and CRY2 proteins from hypothalamus extracts. Equal loading of proteins is demonstrated by GAPDH. **(B)** Hypothalamus CRY1 immunohistochemistry. Paraffin-embedded sections were stained with antibodies against CRY1. Original magnification: ×400. Values are expressed as mean ± standard error of the mean (SEM) of experiments performed in triplicate. **(C)** Expression of CRY1 as detected by immunofluorescence staining. Hypothalamus tissue was stained with an antibody raised against CRY1 (red). DAPI staining (blue) denotes cell nuclei. Values are expressed as mean ± SEM of experiments performed in triplicate. A: control group; B: NMDA group; C: ACTH group; D: MLT group; E: ACTH+MLT group. ^*a*^*P* < 0.05 compared with control group; ^*b*^*P* < 0.05 compared with NMDA group.

We also used immunohistochemical and immunofluorescence staining to investigate the expression of CLOCK, BMAL1, and CRY1 proteins. In the NMDA group, the expression of CLOCK, BMAL1, and CRY1 proteins was significantly lower than that in the control group (*n* = 3) (*P* < 0.05). Furthermore, the expression of CLOCK, BMAL1, and CRY1 proteins in the NMDA+ACTH and NMDA+MLT groups was significantly higher than that in the NMDA group (*P* < 0.05). The increased level of expression was most obvious in the NMDA+ACTH+MLT group relative to the NMDA group (*P* < 0.05). Compared with the control group, the NMDA+ACTH+MLT group showed no significant difference in the immunofluorescence staining experiments (*P* > 0.05), though there was a significantly lower expression of CLOCK protein in the immunohistochemical staining experiments (Figures 4B, 5, 6B,C).

### Expression of Circadian Rhythm Factors Was Positively Correlated With Latency and Negatively Correlated With the Frequency of Flexion Seizures

Spearman's correlation coefficient was used to study the correlations between the expression of circadian rhythm factors and seizure latency and frequency. The expression of circadian rhythm factors was positively correlated with latency to flexion seizure (*P* < 0.001, Table 3) and negatively correlated with the frequency of flexion seizures (Table 3).

**Table 3:** Correlations of circadian rhythm proteins with the latency and frequency of seizures.

**Protein**		**BMAL1**	**CLOCK**	**CRY1**	**CRY2**	**PER1**	**PER2**
Frequency	r	−0.644573	−0.636777	−0.647762	−0.608860	−0.640577	−0.615326
	*P*	<0.001	<0.001	<0.001	<0.001	<0.001	<0.001
Latency	r	0.707102	0.800684	0.712740	0.677922	0.712917	0.669540
	*P*	<0.001	<0.001	<0.001	<0.001	<0.001	<0.001

## Discussion

This study showed that the administration of ACTH and melatonin significantly increased the latency to flexion seizure and significantly reduced the frequency of flexion seizures. Furthermore, NMDA-induced seizures were associated with significant reductions in the expression levels of several key circadian rhythm proteins (CLOCK, BMAL1, PER1, PER2, CRY1, and CRY2). However, the administration of ACTH significantly increased the expression of PER1, CRY1, and CLOCK proteins, while the administration of melatonin significantly increased the expression of all circadian rhythm proteins (CLOCK, BMAL1, PER1, PER2, CRY1, and CRY2). Some of these increases (BMAL1, PER1, PER2, CRY1, and CRY2) were larger in the group of rats receiving a combination of ACTH and melatonin treatments. Finally, we showed that the expression of key circadian rhythm proteins was positively correlated with the latency to flexion seizure and negatively correlated with the frequency of flexion seizures. Collectively, these data demonstrate that these circadian rhythm proteins may play an important role in the pathogenesis of epileptic spasm and demonstrate the anticonvulsant effects of ACTH and melatonin.

In a previous study, Nakken et al. (24) reported that abnormalities of the endogenous circadian rhythm observed in sleep disorders could induce various types of epilepsy, particularly idiopathic generalized epilepsy. Subsequent studies confirmed that the core circadian rhythm genes, *BMAL1* and *CLOCK*, affected both seizure threshold and excitability (13, 25). Furthermore, Wallace et al. (26) reported that BMAL1 and CLOCK, and their respective target genes *PER1* and *PER2*, showed lower levels of expression in the hypothalamus of mice following knockout of the voltage-dependent potassium channel gene KCNA1. In the present study, we found that NMDA-induced seizures were associated with reduced expression of several circadian rhythm proteins (CLOCK, BMAL1, PER1, PER2, CRY1, and CRY2). Collectively, these findings indicate that circadian rhythm factors may be involved in the pathogenesis of epilepsy, including specific forms of epilepsy, such as IS. ACTH, an intermediate product of the HPA axis, is commonly used as the first-line treatment for IS. Previous research has shown that ACTH may regulate the release and expression of neurotransmitters by inhibiting the release of CRH by negative feedback, thus controlling spastic seizures; however, the specific mechanism involved has yet to be elucidated (5). In this study, we found that ACTH treatment reduced the frequency of flexion seizures and increased the expression of PER1, CRY1, and CLOCK proteins.

While numerous researchers have investigated the effect of ACTH on IS, no studies have investigated the effect of melatonin on IS. Melatonin is a hormone secreted by the pineal gland, which is regulated by the circadian rhythm and can also influence the circadian rhythm in turn. Exogenous administration of melatonin is also known to enhance the expression of genes associated with the circadian rhythm (23). Previous studies have also shown that melatonin is associated with epilepsy and that the administration of melatonin can effectively control seizures, increase the threshold of epileptic seizure, and prolong the seizure latency and reduce the severity of epilepsy (27, 28). This study showed that melatonin can reduce the frequency of flexion seizures and increase the expression of CLOCK, BMAL1, PER1, PER2, CRY1, and CRY2 proteins. The combined effect of ACTH and melatonin on the expression of genes associated with the circadian rhythm was more extensive than the effect of either ACTH or melatonin alone, suggesting that circadian rhythm factors may play a key role in the mechanisms underlying the combined anticonvulsant effect of ACTH and melatonin.

It is generally accepted that the circadian rhythm is associated with epilepsy via two principal mechanisms. First, circadian rhythm genes (*BMAL1* and *CLOCK*), and their transcription factor complex, BMAL1–CLOCK, are known to influence the expression of other genes that are causally involved in epilepsy (for example, *PAR DBP, TEF*, and *HLF*) (29, 30). One previous study reported that the knockout of CLOCK in the excitatory pyramidal neurons of rats resulted in a reduced seizure threshold and an increased number of seizures during sleep. This was combined with reduced dendritic spine formation and alterations in the electrophysiological characteristics of the neuronal microcircuits containing excitatory pyramidal cells. These changes resulted in a paroxysmal depolarizing shift, the cellular hallmark of epilepsy (30). The same phenomenon was observed in *BMAL1* knockout mice (29). The second mechanism is related to the mTOR pathway, which is also known to be regulated by circadian rhythm factors (31). Mutations in the mTOR inhibitor genes, *TSC1* and *TSC2*, have been shown to cause overactivity in the mTOR pathway, thus leading to epilepsy in patients with tuberous sclerosis (32). Evidence also suggests that mutations in other regulatory proteins, such as the GATOR1 complex, can lead to disinhibition of the mTOR pathway, thus resulting in nocturnal frontal lobe epilepsy (33). Based on these two theories, previous studies have suggested that key molecules in the mTOR pathway (such as the translation initiation factor 4EBP1 and the kinase S6K1) can change the period and amplitude of CLOCK expression by regulating protein synthesis (34) and that BMAL1 is regulated by the phosphorylation of S6K1 (35). Therefore, circadian rhythm genes maybe involved in the pathogenesis of spasms through the above approaches.

There were several limitations to this study that need to be considered. Firstly, our sample size was small. Secondly, due to funding limitations, we only used an animal model and did not perform clinical studies. Thirdly, this is a acute spasms model, although we speculate that it may have chronic effects, but unable to reflect the actual clinical situation. Finally, we were unable to knock out appropriate genes in order to confirm the mechanisms that may be associated with our observations. Futher experiments and clinical studies are needed to validate the findings of the present study.

## Conclusions

In conclusion, this study showed that NMDA-induced seizures were associated with the reduced expression of circadian rhythm proteins and that the administration of ACTH and/or melatonin increased the latency to seizure and reduced the frequency of flexion seizures. The anticonvulsant effects of ACTH and melatonin are likely to be related to regulatory effects on the expression of genes associated with the circadian rhythm. These results pave the way toward a better understanding of factors associated with the circadian rhythm and their involvement in epilepsy. The findings also enhance our understanding of the mechanisms underlying the anticonvulsant properties of ACTH and melatonin.

## Data Availability Statement

The raw data supporting the conclusions of this article will be made available by the authors, without undue reservation, to any qualified researcher.

## Ethics Statement

The animal study was reviewed and approved by Ethics Committee of the First Medical Center of the PLA General Hospital, China.

## Author Contributions

LW, X-YS, L-PZ, and GY contributed to the conception and design of the study. W-RG, JW, L-YH, LW, and X-YS organized the database. W-RG, Y-LS, SZ, LW, and X-YS performed the statistical analysis. W-RG, LW, and X-YS wrote the first draft of the manuscript. LW, X-YS, W-RG, L-PZ, and GY wrote sections of the manuscript. All authors revised the manuscript and read and approved the submitted version.

## Conflict of Interest

The authors declare that the research was conducted in the absence of any commercial or financial relationships that could be construed as a potential conflict of interest.
